# Supplementary material for: Health-related quality of life and associated factors among family caregivers of patients with cancer in oncologic centers of Northwest Ethiopia
Source: PLoS One. 2024 May 31;19(5):e0304392. doi: 10.1371/journal.pone.0304392 (PMC11142700; doi:10.1371/journal.pone.0304392)
Supplement: S2 Checklist — (DOCX) [file pone.0304392.s002.docx]

**STROBE Checklist**

**Health-related quality of life and associated factors among family caregivers of patients with cancer in oncologic centers of Northwest Ethiopia, a Multicenter cross-sectional study, 2023**

|  | **Item No** | **Recommendation** | **Reported on page No& lines** |
| --- | --- | --- | --- |
| **Title and abstract** | 1 | (*a*) Indicate the study’s design with a commonly used term in the title or the abstract | Used in title and abstract page 2 line 8 |
|  |  | (*b*) Provide in the abstract an informative and balanced summary of what was done and what was found | Page 2\| lines 8-20 |
| **Introduction** | | |  |
| Background/rationale | 2 | Explain the scientific background and rationale for the investigation being reported | Page 3\| lines 2-33  Page 4\| lines 1 |
| Objectives | 3 | State specific objectives, including any prespecified hypotheses | Page 4\|lines 1-3 |
| **Methods** | | |  |
| Study design | 4 | Present key elements of study design early in the paper | Page 4\| line 6 |
| Setting | 5 | Describe the setting, locations, and relevant dates, including periods of recruitment, exposure, follow-up, and data collection | Page 4\|lines 6-9 |
| Participants | 6 | (*a*) Give the eligibility criteria, and the sources and methods of selection of participants | Page 4\|lines 11-17 |
| Variables | 7 | Clearly define all outcomes, exposures, predictors, potential confounders, and effect modifiers. Give diagnostic criteria, if applicable | Page 5\|lines 13 – 19 |
| Data sources/ measurement | 8* | For each variable of interest, give sources of data and details of methods of assessment (measurement). Describe comparability of assessment methods if there is more than one group | Page 6\|lines 5 – 31  Page 7\|lines 1-20 |
| Bias | 9 | Describe any efforts to address potential sources of bias | Page 7\|lines 21-27 |
| Study size | 10 | Explain how the study size was arrived at | Page 4\|lines 19- 26 |
| Quantitative variables | 11 | Explain how quantitative variables were handled in the analyses. If applicable, describe which groupings were chosen and why | Page 7\|lines 30 -31  Page 8\|line 1 |
| Statistical methods | 12 | (*a*) Describe all statistical methods, including those used to control for confounding | Page 7\| lines 13-20 |
|  |  | (*b*) Describe any methods used to examine subgroups and interactions | N/A |
|  |  | (*c*) Explain how missing data were addressed | N/A |
|  |  | (*d*) If applicable, describe analytical methods taking account of sampling strategy | N/A |
|  |  | (*e*) Describe any sensitivity analyses | N/A |
| **Results** | | |  |
| Participants | 13* | (a) Report numbers of individuals at each stage of study—eg numbers potentially eligible, examined for eligibility, confirmed eligible, included in the study, completing follow-up, and analysed | N/A |
|  |  | (b) Give reasons for non-participation at each stage | N/A |
|  |  | (c) Consider use of a flow diagram | N/A |
| Descriptive data | 14* | (a) Give characteristics of study participants (eg demographic, clinical, social) and information on exposures and potential confounders | N/A |
|  |  | (b) Indicate number of participants with missing data for each variable of interest |  |
| Outcome data | 15* | Report numbers of outcome events or summary measures | N/A |
| Main results | 16 | (*a*) Give unadjusted estimates and, if applicable, confounder-adjusted estimates and their precision (eg, 95% confidence interval). Make clear which confounders were adjusted for and why they were included | N/A |
|  |  | (*b*) Report category boundaries when continuous variables were categorized | Page 9-10\|Table 1  Page 10-11\|Table 2 |
|  |  | (*c*) If relevant, consider translating estimates of relative risk into absolute risk for a meaningful time period | N/A |
| Other analyses | 17 | Report other analyses done—eg analyses of subgroups and interactions, and sensitivity analyses | N/A |
| **Discussion** | | |  |
| Key results | 18 | Summarise key results with reference to study objectives | Page 15\| lines 9-16 |
| Limitations | 19 | Discuss limitations of the study, taking into account sources of potential bias or imprecision. Discuss both direction and magnitude of any potential bias | Page 16\| lines 31 – 32  Page 17\|lines 1-7 |
| Interpretation | 20 | Give a cautious overall interpretation of results considering objectives, limitations, multiplicity of analyses, results from similar studies, and other relevant evidence | Page 15\| lines 17-26  Page 16\| lines 1- 29 |
| Generalisability | 21 | Discuss the generalisability (external validity) of the study results | Page 17\|lines 9-16 |
| **Other information** | | |  |
| Funding | 22 | Give the source of funding and the role of the funders for the present study and, if applicable, for the original study on which the present article is based | Page 17\| line 23 |

*Give information separately for exposed and unexposed groups.
