# Supplementary material for: Health-related quality of life and associated factors among family caregivers of patients with cancer in oncologic centers of Northwest Ethiopia
Source: PLoS One. 2024 May 31;19(5):e0304392. doi: 10.1371/journal.pone.0304392 (PMC11142700; doi:10.1371/journal.pone.0304392)
Supplement: Attachment — Submitted filename: PONE-D-24-10340_reviewer.pdf [file pone.0304392.s005.pdf]

PLOS ONE

Health-related quality of life and associated factors among family caregivers of patients with cancer in oncologic centers of Northwest Ethiopia, a Multicenter cross-sectional study, 2023

--Manuscript Draft--

**Manuscript Number:**	PONE-D-24-10340
**Article Type:**	Research Article
**Full Title:**	Health-related quality of life and associated factors among family caregivers of patients with cancer in oncologic centers of Northwest Ethiopia, a Multicenter cross-sectional study, 2023
**Short Title:**	Health-related quality of life and associated factors among family caregivers of patients with cancer
**Corresponding Author:**	Fasil Bayafers Tamene<br>Debre Markos University<br>Debre Markos, Amhara ETHIOPIA
**Keywords:**	Health related quality of life, Cancer, Caregivers, Depression, Anxiety, Social support
**Abstract:**	Background: Providing care for individuals dealing with long-term illnesses like cancer demands significant amounts of time, energy, and emotional investment, potentially resulting in a challenging and overwhelming quality of life for those providing the care.<br>Objective: The aim of this study was to assess the level of health-related quality of life (HRQoL) and associated factors among family caregivers of patients with cancer in oncologic centers of Northwest Ethiopia.<br>Method: A cross-sectional study was conducted among 412 family caregivers of patients with cancer who were following treatment at oncologic centers in Northwest Ethiopia from August to October 2023. Study participants were enrolled using systematic random sampling. Data entry and analysis were done using Epi-data version 4.6.1 and SPSS version 26, respectively. Linear regression was used to assess the association between quality of life and independent variables. Variables with a p-value < 0.05 at a 95% confidence interval were taken as statistically significant.<br>Result: Out of a total of 422 approached samples, 412 eligible caregivers were included in the study, resulting in a response rate of 97.6%. The mean score of the overall Quality of Life Brief - Scale Version was 52.7 ± 9.57. Being Spouse (β= -3.39; 95% CI: -6.49, -0.29), presence of chronic illness (β = -3.43; 95% CI: -5.56, -1.31), depression, (β = -2.55; 95% CI: -4.34, -0.75), anxiety (β = -3.27; 95% CI: -5.22, -1.32), and social support, (β = -3.61; 95% CI: -6.20, -1.02) were negatively associated with quality of life.<br>Conclusion and recommendation: In this study, the psychological domain was recorded as having the lowest mean score. Caregivers being as spouse, who were with chronic illness, manifested depression and anxiety and had a poor social support needs attention and support to improve QoL.
**Order of Authors:**	Fasil Bayafers Tamene
	Endalamaw Aschale Mihiretie
	Akalu Fetene Desalew
	Fasika Argaw Tafesse
	Samuel Agegnew Wondm
**Additional Information:**	
**Question**	**Response**
**Financial Disclosure**<br><br>Enter a financial disclosure statement that describes the sources of funding for the	The author(s) received no specific funding for this work.

Powered by Editorial Manager® and ProduXion Manager® from Aries Systems Corporation

work included in this submission. Review the submission guidelines for detailed requirements. View published research articles from *PLOS ONE* for specific examples.<br><br>This statement is required for submission and **will appear in the published article** if the submission is accepted. Please make sure it is accurate.<br><br>**Funded studies**<br>Enter a statement with the following details:<br>• Initials of the authors who received each award<br>• Grant numbers awarded to each author<br>• The full name of each funder<br>• URL of each funder website<br>• Did the sponsors or funders play any role in the study design, data collection and analysis, decision to publish, or preparation of the manuscript?<br><br>Did you receive funding for this work?	
**Competing Interests**<br><br>Use the instructions below to enter a competing interest statement for this submission. On behalf of all authors, disclose any competing interests that could be perceived to bias this work—acknowledging all financial support and any other relevant financial or non-financial competing interests.<br><br>This statement is required for submission and **will appear in the published article** if the submission is accepted. Please make sure it is accurate and that any funding sources listed in your Funding Information later in the submission form are also declared in your Financial Disclosure statement.<br><br>View published research articles from *PLOS ONE* for specific examples.	The authors have declared that no competing interests exist.

**NO authors have competing interests**<br><br>Enter: *The authors have declared that no competing interests exist.*<br><br>**Authors with competing interests**<br><br>Enter competing interest details beginning with this statement:<br><br>*I have read the journal's policy and the authors of this manuscript have the following competing interests: [insert competing interests here]*<br><br>* typeset	
**Ethics Statement**<br><br>Enter an ethics statement for this submission. This statement is required if the study involved:<br><br>• Human participants<br>• Human specimens or tissue<br>• Vertebrate animals or cephalopods<br>• Vertebrate embryos or tissues<br>• Field research<br><br>Write "N/A" if the submission does not require an ethics statement.<br><br>General guidance is provided below. Consult the submission guidelines for detailed instructions. **Make sure that all information entered here is included in the Methods section of the manuscript.**	The study obtained ethical approval from the institutional review board of the College of Medicine and Health Sciences at Bahir Dar University, with reference number 796/2023.

Powered by Editorial Manager® and ProduXion Manager® from Aries Systems Corporation

**Format for specific study types**<br><br>**Human Subject Research (involving human participants and/or tissue)**<br>• Give the name of the institutional review board or ethics committee that approved the study<br>• Include the approval number and/or a statement indicating approval of this research<br>• Indicate the form of consent obtained (written/oral) or the reason that consent was not obtained (e.g. the data were analyzed anonymously)<br><br>**Animal Research (involving vertebrate animals, embryos or tissues)**<br>• Provide the name of the Institutional Animal Care and Use Committee (IACUC) or other relevant ethics board that reviewed the study protocol, and indicate whether they approved this research or granted a formal waiver of ethical approval<br>• Include an approval number if one was obtained<br>• If the study involved *non-human primates*, add *additional details* about animal welfare and steps taken to ameliorate suffering<br>• If anesthesia, euthanasia, or any kind of animal sacrifice is part of the study, include briefly which substances and/or methods were applied<br><br>**Field Research**<br><br>Include the following details if this study involves the collection of plant, animal, or other materials from a natural setting:<br>• Field permit number<br>• Name of the institution or relevant body that granted permission	
**Data Availability**<br><br>Authors are required to make all data underlying the findings described fully available, without restriction, and from the time of publication. PLOS allows rare exceptions to address legal and ethical concerns. See the PLOS Data Policy and FAQ for detailed information.	Yes - all data are fully available without restriction

A Data Availability Statement describing where the data can be found is required at submission. Your answers to this question constitute the Data Availability Statement and **will be published in the article**, if accepted.<br><br>**Important:** Stating 'data available on request from the author' is not sufficient. If your data are only available upon request, select 'No' for the first question and explain your exceptional situation in the text box.<br><br>Do the authors confirm that all data underlying the findings described in their manuscript are fully available without restriction?	
**Describe where the data may be found in full sentences. If you are copying our sample text, replace any instances of XXX with the appropriate details.**<br><br>• If the data are **held or will be held in a public repository**, include URLs, accession numbers or DOIs. If this information will only be available after acceptance, indicate this by ticking the box below. For example: *All XXX files are available from the XXX database (accession number(s) XXX, XXX.).*<br>• If the data are all contained **within the manuscript and/or Supporting Information files**, enter the following: *All relevant data are within the manuscript and its Supporting Information files.*<br>• If neither of these applies but you are able to provide **details of access elsewhere**, with or without limitations, please do so. For example:<br><br>*Data cannot be shared publicly because of [XXX]. Data are available from the XXX Institutional Data Access / Ethics Committee (contact via XXX) for researchers who meet the criteria for access to confidential data.*<br><br>*The data underlying the results presented in the study are available from (include the name of the third party*	The datasets used and analyzed during the current study are available.

and contact information or URL).<br>• This text is appropriate if the data are owned by a third party and authors do not have permission to share the data.<br><br>* typeset	
Additional data availability information:	

Manuscript Click here to access/download;Manuscript;Manuscript.docx

Health-related quality of life and associated factors among family caregivers of patients with cancer in oncologic centers of Northwest Ethiopia, a Multicenter cross-sectional study, 2023

Fasil Bayafers Tamene[1*], Endalamaw Aschale Mihiretie[2], Akalu Fetene Desalew[1], Fasika Argaw Tafesse[3], Samuel Agegnew Wondm[1]

[1]Department of Pharmacy, College of Medicine and Health Science, Debre Markos University, Debre Markos, Ethiopia

[2]Department of Pharmacy, College of Medicine and Health Science, Bahir Dar University, Bahir Dar, Ethiopia

[3]Department of Pharmacy, College of Medicine and Health Science, Dilla University, Dilla, Ethiopia

***Correspondence:** Fasil Bayafers Tamene

E-mail: fasil.baya@gmail.com

Abstract

Background: Providing care for individuals dealing with long-term illnesses like cancer demands significant amounts of time, energy, and emotional investment, potentially resulting in a challenging and overwhelming quality of life for those providing the care.

Objective: The aim of this study was to assess the level of health-related quality of life (HRQoL) and associated factors among family caregivers of patients with cancer in oncologic centers of Northwest Ethiopia.

Method: A cross-sectional study was conducted among 412 family caregivers of patients with cancer who were following treatment at oncologic centers in Northwest Ethiopia from August to October 2023. Study participants were enrolled using systematic random sampling. Data entry and analysis were done using Epi-data version 4.6.1 and SPSS version 26, respectively. Linear regression was used to assess the association between quality of life and independent variables. Variables with a p-value < 0.05 at a 95% confidence interval were taken as statistically significant.

Result: Out of a total of 422 approached samples, 412 eligible caregivers were included in the study, resulting in a response rate of 97.6%. The mean score of the overall Quality of Life Brief - Scale Version was 52.7 ± 9.57. Being Spouse (β= -3.39; 95% CI: -6.49, -0.29), presence of chronic illness (β = -3.43; 95% CI: -5.56, -1.31), depression, (β = -2.55; 95% CI: -4.34, -0.75), anxiety (β = -3.27; 95% CI: -5.22, -1.32), and social support, (β = -3.61; 95% CI: -6.20, -1.02) were negatively associated with quality of life.

Conclusion and recommendation: In this study, the psychological domain was recorded as having the lowest mean score. Caregivers being as spouse, who were with chronic illness, manifested depression and anxiety and had a poor social support needs attention and support to improve QoL.

Keywords: Health related quality of life, Cancer, Caregivers, Depression, Anxiety, Social support

Introduction

Cancer is regarded as the prominent ailment of the modern era, being a primary contributor to global mortality and morbidity. Despite advancements in diagnosis and treatment, its cure remains elusive. According to the World Health Organization (WHO), cancer ranks among the foremost causes of death from disease. The International Agency for Research on Cancer projected that in 2020 alone, there were 18.1 million diagnosed cases globally, with this number anticipated to rise to 27 million over the next two decades [1].

In low-income nations with restricted logistical resources, cancer is becoming a significant obstacle to maintaining citizens' quality of life [2]. Like other chronic illnesses, cancer presents various complications and can limit self-reliance, necessitating assistance from caregivers within the patient's close network, often referred to as informal or non-professional caregivers. While these caregivers play a crucial role in aiding and comforting cancer patients, they often bear a substantial burden across multiple domains, which can profoundly affect their own quality of life [3,4].

The World Health Organization (WHO) has defined QOL as "individuals' perceptions of their position in life in the context of the culture and value systems in which they live and in relation to their goals, expectations, standards and concerns [5]." The profound impact of cancer extends beyond patients to their families [6]. Providing care for individuals with long-term illnesses such as cancer demands significant time, effort, and emotional investment, potentially resulting in a highly stressful and burdensome quality of life for caregivers [7]. Furthermore, the effect of cancer on the health-related quality of life (HRQOL) of family caregivers can hinder their capacity to effectively care for and support the patients [8]. A multitude of factors affected the QOL of family caregivers of adult cancer patients. These
comma here
may include sociodemographic factors such as age, gender, place of residence marital status, religion, educational level, occupation, monthly income, family size, types of relation to a patient and caregiving duration [9-16] and clinical social support related variables of caregivers and patients such as presence of chronic illness, depression, anxiety, level of social support, type of cancer, hospital admission of the patients, type of treatment and performance status [17-23].

In our country, caregiving for an ill family member holds deep cultural significance. Understanding the importance of health-related quality of life (HRQoL) as a health measure, it becomes essential to investigate the factors impacting caregivers' HRQoL. In economically challenged nations like Ethiopia, there is limited knowledge about the HRQoL of family

caregivers of adult cancer patients. Therefore, the aim of this study was to assess the level of health-related quality of life and associated factors among family caregivers of patients with cancer in oncologic centers of Northwest Ethiopia.

Methods

Study setting, period and design

A cross sectional study was conducted at the University of Gondar Comprehensive Specialized Hospital (UoGCSH), Felege-Hiwot Comprehensive Specialized Hospital (FHCSH), and Tibebe-Ghion Comprehensive Specialized Hospital (TGCSH) from August to October 2023.

Population, inclusion and exclusion criteria

All family caregivers of patients with cancer at the oncologic centers of the northwest Ethiopia were the source population. All family caregivers of cancer patients at UoGCSH, FHCSH and TGCSH who fulfills the inclusion criteria during the data collection period were study population. Caregivers with the age of 18 years and above, caring for patients with definitive diagnosis of cancer and those who can be identified by the patient as being caregivers was included in the study. Caregivers with psychiatric conditions and those who were unwilling to participate were excluded from the study.

Sample size determination

Single population proportion formula was used to determine the number of participants needed for this study. To get better representative sample, we used proportion as 50% in sample size calculation. $n = \frac{(Z\alpha/2)^2 \times p\,(1-p)}{d^2}$, where n is the desired sample size for a population of >10,000, Z is the typical normal distribution set at 1.96 (which corresponds to 95% CI), the p-value signifies that positive prevalence was utilized in calculating the optimal sample size, and W is the degree of accuracy 0.05 required (a marginal error is 0.05). Then computing for $n = 1.96^{2*}0.5(1-0.5)/0.05^2$, n = 384. By adding 10% non-response rate, the final calculated sample size was 422.

Sampling technique and procedure

The total number of cancer patients over a three-month period was extracted from patient registration documents to allocate respondents proportionally across study areas. Following proportional allocation, a systematic random sampling method was employed to choose study participants. The sampling fraction (k) was determined by dividing the total number of cancer patients in each study area by the overall sample size; (925/422 equals 2.19, approximated to 2). Proportional allocation was then calculated for each study area (UOGCSH: 422x358/925=163, FHCSH: 422x376/925 =172, TGCSH: 422*191/925=87). A starting point was randomly selected from 1 or 2. Subsequently, participants were interviewed, and relevant data was simultaneously gathered from medical charts for every two patients until the required sample size was achieved. A unique patient identification card number was assigned as a questionnaire code to prevent duplicate inclusion of the same patient in the study.

Study variables

Health-related quality of life was the main outcome variable. The independent variables were the sociodemographic characteristics of the participants (like sex, age, marital status, educational status, occupation, monthly income, patient's health insurance status, relationship to patient, family size, duration of caregiving and support from other family members) and clinical related variables (i.e. presence of chronic disease of caregiver, presence of substance use, depression, anxiety, and eastern cooperative oncology group performance status) and level of social support.

Operational definitions

Anxiety: Individuals who score ≥10 from GAD-7 were considered as having anxiety [24].

Depression: Individuals who are found to score≥10 from PHQ-9 were considered as having depression [25].

Health related quality of life: Based on the WHOQOL-BREF 26 items, an individuals with the score approaches to 100 indicates the best possible quality of life, and individuals with the score approaches to zero indicates the poorest quality of life [26].

Social support: According to the Oslo-3 social support scale which ranges from 3 to 14, individuals who score between 3 and 8 are considered to have poor social support, who score of 9– 11 is considered as having moderate social support, and score of 12– 14 is considered as having strong social support [27]

Substance use: current users: using at least one of a specific substance (alcohol, Khat or cigarettes) for nonmedical purposes within the last 3 months, according to the alcohol, smoking, and substance involvement screening tool (ASSIST) [28].

Data collection instrument, procedures, and quality control

A structured questionnaire, based on existing literature [29], underwent adaptations to suit the study area's context and the socio-demographic characteristics of the participants. This questionnaire was translated into the local Amharic language and subsequently back-translated into English to ensure consistency. Variables obtained from patients' medical records did not necessitate translation. The data collected by interviewing family caregivers includes sociodemographic characteristics, presence of chronic disease, substance use, anxiety, depression and social support. The patients' medical charts were used to fill in clinically related variables like cancer diagnosis of the patient, duration of cancer diagnosis and treatment, current treatment modality, functional status and metastasis.

The data collection tool has seven parts. The first part contains socio-demographic characteristics of the study participants like age, sex, marital status, monthly income, residence, religion, educational status, occupation, patient's health insurance status, relationship to patient, duration of caregiving, care support from other family members, duration of caregiving per month and number of family size. The second section consisted of clinical related variables like presence of chronic disease, presence of substance use, depression and anxiety of caregiver, cancer diagnosis of the patient, duration of cancer diagnosis and treatment, current treatment modality, ECOG (Eastern Cooperative Oncology Group), and presence of metastasis.

The third section consisted HRQoL measuring tool. Health related quality of life was assessed by utilizing the World Health Organization Quality of Life Scale–Brief version (WHOQOL–BREF) which is a 26-item self-administered generic questionnaire. The WHOQOL-BREF is a sound, cross-culturally valid assessment of QoL, as indicated by its
What is this number for?
four domains: physical, psychological, social, and environment [30] (37). It creates a profile with four domain scores: physical health (7 items), psychological health (6 items), social relationships (3 items), and environmental domain (8 items) as well as two individually scored items concerning the individuals' impression of their quality of life (QI) and health (Q2). The scores of items in each domain will be combined, and the mean or median will be

calculated to produce the domain score [31]. WHOQOL–BREF has been used in Ethiopia [32].

The fourth section of the questionnaire is assessing social support by the Oslo-3 social support scale. The OSS-3 scores ranged from 3–14, with a score of 3–8 = poor social support; 9–11 = moderate social support; and 12–14 = strong social support [27]. The fifth section involved the current substance use assessment tool. ASSIST was utilized to screen participants briefly for the use of psychoactive substances. This tool was developed and validated by the WHO [28].

The sixth section consisted assessment of depression by using the (**PHQ-9).** The PHQ-9 score ranges from 0 to 27. Each of the 9 items was scored from 0 (not at all) to 3 (nearly every day). A PHQ-9 score 0-4 indicates minimal/no depression, 5-9 indicates mild depression, 10–14 indicates moderate depression, 15–19 indicates moderately severe depression and a score of 20 to 27 indicates severe type of depression [33]. Moreover, PHQ-9 has been validated in Ethiopian healthcare context with specificity and sensitivity of 67% and 86%, respectively. A cut-off point of 10 or more has been used to screen for depression [25].

The seventh section of the questionnaire assessed anxiety by using **GAD-7**. Scores of 0, 1, 2, and 3 were assigned to the response categories of "not at all," "several days," "more than half the days," and "nearly every day," respectively, and the scores for the seven questions were added together. Using the threshold score of 10, the GAD-7 has a sensitivity of 89% and a specificity of 82% for GAD [24].

Data collection involved face-to-face interviews utilizing a pretested and structured questionnaire. At UoGCSH and FHCSH, three nurses with bachelor's degrees, supervised by a senior oncology nurse holding a master's degree, conducted the interviews. Similarly, at TGCSH, two nurses with bachelor's degrees, overseen by a senior oncology nurse with a bachelor's degree, conducted the interviews. Each day, supervisors provided necessary supplies to the data collectors and ensured the accuracy of completed questionnaires, addressing any issues promptly. The lead researcher provided resources for all study areas.

Data entry and analysis

The collected data underwent cleaning, coding, and entry into Epi Data 4.6.0, followed by analysis using Statistical Package for Social Studies (SPSS) version 26. Descriptive analysis utilized measures such as mean with standard deviation (SD), frequency, and percentages to

assess data distribution. Bivariate and multivariate linear regression analyses were conducted to explore the relationship between HRQoL and independent variables.

Linearity was confirmed via scatter plots, demonstrating a negative association between HRQoL and all continuous independent variables. Normality assumption was verified through histograms and normal probability-probability (P-P) plots, indicating a normal distribution with skewness ranging between ±1, signifying normality. The Durbin-Watson statistic ranged from 1.5 to 2.5, affirming independent observations. Multicollinearity was assessed, with the maximum Variance Inflation Factor (VIF) found to be less than 5, indicating acceptability.

Variables with a p-value < 0.25 in simple linear regression were chosen for multiple linear regressions. A p-value < 0.05 was considered indicative of an independently associated factor in multivariable linear regression. Model fitness was evaluated, with statistical significance observed at F = 5.13, P value < 0.001, R = 0.681, R^2 = 0.431, and adjusted R^2 = 0.357. Unstandardized beta coefficients with a 95% confidence interval (CI) were utilized to assess the level of association and statistical significance in multiple linear regression analysis

Ethics approval and consent to participate

The study obtained ethical approval from the institutional review board of the College of Medicine and Health Sciences at Bahir Dar University, with reference number 796/2023. Prior to their participation, all study participants were fully informed about the study's objectives and provided written consent. Measures were taken to ensure participants' privacy, and personal identifiers were omitted from the data collected. The study adhered to the principles outlined in the Declaration of Helsinki.

Results

Sociodemographic characteristics among family caregivers of cancer patients

Out of a total of 422 individuals approached, 412 eligible caregivers were enrolled in the study, resulting in a response rate of 97.6%. The majority (51.9%) were female, with an average age of 36.1 (±12.2) years. Nearly half (48.3%) of the participants were married, and more than two-thirds (72.6%) resided in urban areas. About a third (31.6%) of respondents

had completed secondary school education, and a similar proportion (34.7%) were employed in the private sector. Close to half (45.9%) of participants reported a monthly income of less than 3000 ET birr, while over two-thirds (67.2%) had health insurance coverage. Regarding their relationship to the patient, more than a third (34.7%) were spouses, and over half (55.3%) had families consisting of 4 to 7 members. Approximately two-thirds (64.3%) of participants had been providing care for a duration ranging from 12 to 36 months, and nearly all (90.5%) received support from other family members (Table 1).

Table 1. Sociodemographic characteristics among family caregivers of cancer patients (n = 412)

Variables	Categories	Frequency	Percentage
Sex	Male	198	48.1
	Female	214	51.9
Age	18-28	77	18.7
	29-39	162	39.3
	40-50	104	25.2
	51-60	58	14.1
	>60	11	2.7
Marital status	Single	168	40.8
	Married	199	48.3
	Divorced	26	6.3
	Widowed	19	4.6
Residence	Urban	299	72.6
	Rural	113	27.4
Religion	Orthodox	330	80.1
	Muslim	70	17.0
	Protestant/Catholic	12	2.9
Educational level	No formal education	26	6.3
	Primary (1–8 grades)	75	18.2
	Secondary (9–12 grade)	130	31.6
	Diploma and above	181	43.9
Occupation	Farmer	29	7.0
	Government employee	79	19.2
	Private employee	143	34.7
	Student	36	8.7
	Jobless	73	17.7
	Housewife	52	12.6
Monthly income in birr	<3000 ET. birr	189	45.9
	3001-7000 ET. birr	166	40.3
	7001-10000 ET birr	33	8.0

	>10000 ET. birr	24	5.8
Health insurance	Yes	277	67.2
	No	135	32.8
Relationship to patient	Spouse	143	34.7
	Parent	54	13.1
	Son/daughter	135	32.8
	Brother/sister	80	19.4
Family size	≤3	39	9.5
	4-7	228	55.3
	≥8	145	35.2
Duration of care giving (per month)	< 12 months	66	16.0
	12-36 Months	265	64.3
	> 36 months	81	19.7
Care support from other family member	Yes	373	90.5
	No	39	9.5
Family history of cancer	Yes	60	14.6
	No	352	85.4

General characteristics among cancer patients

The majority of patients (60.2%) were female, with an average age of 41.91 (±16.5) years. Around a quarter (24.0%) had been diagnosed with breast cancer, and most of them (61.7%) had been living with the illness for less than a year. Over half (58.5%) had undergone treatment for less than a year, with the majority (63.3%) receiving chemotherapy. A quarter (25.0%) had an ECOG score of III, and more than a third (36.7%) had cancer that had metastasized. About one-seventh (14.6%) reported a family history of cancer (Table 2).

Table 2. General characteristics of cancer patients (n = 412)

Variables	Categories	Frequency	Percent
Sex	Male	164	39.8
	Female	248	60.2
Age	41.91 (±16.5)*		
Cancer type	Breast	99	24.0
	Cervical	86	20.9
	Colorectal	53	12.9
	Lymphoma	34	8.3
	Ovarian	32	7.8
	Lung	30	7.3

	Bone and soft tissue	28	6.8
	Head and neck	21	5.1
	other**	29	7.0
Duration of illness	< 1 year	254	61.7
	1-5 year	141	34.2
	>5 year	17	4.1
Duration of Treatment	< 1 year	241	58.5
	1-5 year	162	39.3
	>5 year	9	2.2
Type of treatment	Chemotherapy	261	63.3
	Surgery	39	9.5
	Chemotherapy and surgery	112	27.2
Functional status (ECOG)	I	152	36.9
	II	138	33.5
	III	103	25.0
	IV	19	4.6
Metastasis	Yes	151	36.7
	No	261	63.3

*, Mean and standard deviation; **, Hodking lymphoma, Pancreatic cancer, Squamous cell carcinoma, Multiple myeloma, Hepato-cellular carcinoma

Clinical, substance and psychological related characteristics among family caregivers of cancer patients

Regarding clinical variables, about a quarter (25.2%) of participants had chronic illnesses, with diabetes mellitus being the most common. Nearly one-third (31.1%) reported using psychoactive substances, primarily alcohol. A significant portion (42.5%) experienced symptoms of depression, and close to one-third (30.6%) reported experiencing anxiety. Additionally, over half (55.1%) of participants reported poor social support (Table 3).

Table 3. Clinical, substance and psychological related characteristics among caregivers of cancer patients (n = 412)

**Variables**	**Categories**	**Frequency**	**Percent**
Presence of chronic illness*	yes	104	25.2
	No	308	74.8
Substance use	Yes	128	31.1
	No	284	68.9
Type of substance use	Alcohol	89	69.6
	Khat	26	20.3
	Cigarette	13	10.1
Depression	Yes	175	42.5

	No	237	57.5
Anxiety	Yes	126	30.6
	No	286	69.4
Social support	Poor	227	55.1
	Moderate	124	30.1
	Strong	61	14.8

* Hypertension, DM, Cardiac disorder, Respiratory disorder, Arthritis, PUD, HIV

Self-rated perceived quality of life and health satisfaction among family caregivers of cancer patients

Over a third (38.6%) of respondents indicated that they perceived their HRQoL as poor. In terms of health satisfaction, around one-third (33.7%) reported dissatisfaction, while slightly more than a quarter (29.0%) expressed neither satisfaction nor dissatisfaction (Table 4).

Table 4. Self-rated perceived quality of life and health satisfaction among family caregivers of cancer patients (n = 412)

**Variable**	**Category**	**Frequency (%)**
Perceived quality of life	Very poor	44 (10.7)
	Poor	159 (38.6)
	Neither poor nor good	141 (34.2)
	Good	48 (11.7)
	Very good	20 (4.9)
Perceived health satisfaction	Very dissatisfied	74(18.0)
	dissatisfied	139 (33.7)
	Neither satisfied nor dissatisfied	120 (29.1)
	Satisfied	42 (10.2)
	Very satisfied	37 (9.0)

Health related quality of life among family caregivers of cancer patients

In this study, the average score for the quality of life among family caregivers of cancer patients was 52.7 (95% CI: 51.70, 53.63), with a standard deviation of 9.57. The range for the overall quality of life scores among participants varied from 26.5 to 81.5. Across the four

domains of quality of life, participants had the lowest average score in the psychological domain (47.82±16.61) and the highest mean score in the environmental domain (58.30±17.73) (Table 5).

Table 5. Health related quality of life among caregivers of cancer patients (n = 412)

Domains	Mean ± SD	95% CI	Percent of participants who scored below the mean
Physical	53.25±21.72	(50.94, 55.39)	54.4
Psychological	47.82±16.61	(46.18, 49.43)	63.1
Social relationship	51.44±19.48	(49.64, 53.48)	54.9
Environmental	58.30±17.73	(56.51, 60.14)	42.2
Overall HRQoL	52.70±9.57	(51.70, 53.63)	40.4

Factors associated with health related quality of life among family caregivers of cancer patients

After adjusting for multiple linear regression analysis, some variables were found to have a significant association with HRQoL. Accordingly, participants those relations were as spouse had 3.39 times lower overall HRQoL than those relations to the patient was as brother or sister (B = -3.39; 95% CI: -6.49, -0.29). Concerning the presence of chronic illness, respondents with chronic illness had 3.43 times lower overall HRQoL than those without chronic illness (β = -3.43; 95% CI: -5.56, -1.31). In terms of experiencing depression, participants who manifested depression had 2.55 times lower overall HRQoL than those who did not manifested (β = -2.55; 95% CI: -4.34, -0.75). Similarly, patients who developed anxiety had 3.27 times decreased overall HRQoL than those without anxiety (β = -3.27; 95% CI: -5.22, -1.32). Regarding social support, caregivers who got a poor social support had decreased overall HRQoL by 3.61 times than those who got strong social support (β = -3.61; 95% CI: -6.20, -1.02) (Table 6).

Table 6. Multivariate linear regression for overall quality of life and associated factors among family caregivers of cancer patients (n = 412)

Variables	Categories	SLR: β (95%CI)	MLR: β (95%CI)	P-value
Sex	Male	-1.32 (-3.17, 0.52)	0.52 (-1.80, 2.84)	0.661
	Female	1	1	
Age	>60	5.54 (-0.51, 11.59)	3.70 (-2.21, 9.62)	0.219
	51-60	-0.87 (-4.14, 2.38)	-0.81 (-4.08, 2.46)	0.627
	40-50	1.50 (-1.31, 4.32)	0.70 (-2.11, 3.51)	0.625

	29-39	1.17 (-1.42, 3.76)	0.31 (-2.21, 2.83)	0.807
	18-28	1	1	
Marital status	Single	0.20 (-1.77, 2.17)	2.12 (-.28, 4.53)	0.084
	Divorced	0.13 (3.79, 4.05)	1.55 (-2.30, 5.42)	0.428
	Widowed	-3.59 (-8.00, 0.82)	-3.67 (-7.95, 0.60)	0.092
	Married	1	1	
Educational level	No formal education	2.54 (-1.38, 6.48)	3.07 (-0.68, 6.84)	0.109
	Primary (1–8 grades)	0.33 (-2.33, 3.01)	1.21 (-1.35, 3.79)	0.353
	Secondary (9–12 grade)	0.99 (-3.13, 1.14))	0.02 (-2.02, 2.07)	0.982
	Diploma and above	1	1	
Occupation	Farmer	1.01 (-3.06, 5.08)	-0.62 (-4.55, 3.29)	0.753
	Private employee	0.78 (-1.82, 3.39)	-0.06 (-2.57, 2.45)	0.960
	Student	-1.01 (-4.77, 2.76)	-0.73 (-4.36, 2.89)	0.691
	Jobless	-1.89 (-4.94, 1.14)	-0.79 (-3.84, 2.25)	0.610
	Housewife	-0.41 (-3.73, 2.91)	-2.14 (-5.39,1.10)	0.196
	Government employee	1	1	
Monthly income in birr	≤ 3000 ET. birr	-2.59 (-6.46, 1.26)	-1.97 (-5.85,1.91)	0.319
	3001-7000 ET. birr	1.02 (-2.87, 4.92)	1.148 (-2.77, 5.07)	0.565
	7001-10000 ET birr	0.61 (-4.25, 5.48)	2.37 (-2.43, 7.17)	0.333
	>10000 ET. birr	1	1	
Health insurance	No	-1.31 (-3.28, 0.66)	-0.62 (-2.62, 1.37)	0.540
	Yes	1	1	
Relationship to patient	Spouse	-2.92 (-5.53, -0.41	-3.39 (-6.49, -0.29)*	**0.032**
	Parent	2.22 (-0.99, 5.44)	0.59 (-2.55, 3.73)	0.711
	Son/daughter	0.37 (-2.23, 2.98)	-0.22 (-2.79, 2.34)	0.866
	Brother/sister	1	1	
Family size	≤3	0.05 (-3.32, 3.34)	-0.15 (3.46, 3.15)	0.927
	4-7	2.43 (0.44, 4.42)	1.67 (-0.30, 3.64)	0.097
	≥8	1	1	
Duration of care giving (per month)	> 36 months	-3.36 (-6.44, -0.27)	-2.36 (-5.37, 0.64)	0.123
	12-36 months	0.51 (-2.05, 3.07)	-0.63 (-3.23, 1.97)	0.633
	< 12 months		1	
Care support from other family member	No	-2.55 (-5.72, 0.60)	-2.69 (-5.82, 0.43)	0.091
	Yes	1	1	
Presence of chronic illness	Yes	-5.17 (-7.24, -3.09)	-3.43 (-5.56, -1.31)*	**0.002**
	No	1	1	
Substance use	Yes	-1.25 (-3.25, 0.75)	-0.56 (-2.53, 1.41)	0.577
	No	1	1	
Depression	Yes	-2.95 (-4.80, -1.09)	-2.55 (-4.34, -0.75)*	**0.005**
	No	1	1	

Anxiety	Yes	-4.24 (-6.21, -2.27)	-3.27 (-5.22, -1.32)*	**0.001**
	No	1	1	
Social support	Poor	-3.49 (-6.18, -0.81)	-3.61 (-6.20, -1.02)*	**0.006**
	Moderate	-0.54 (-3.45, 2.36)	-1.35 (-4.12, 1.42)	0.338
	Strong	1		
ECOG	IV	-0.87 (-5.45, 3.71)	-0.87 (-5.19, 3.44)	0.691
	III	-1.43 (-3.38, 0.97)	-1.28 (-3.59, 1.03)	0.276
	II	-1.02 (-3.23, 1.19)	-0.86 (-2.95, 1.21)	0.414
	I	1	1	

R = 0.681, R^2 = 0.431, and adjusted R^2 = 0.357

*P<0.05; ß, unstandardized beta coefficient; MLR, multiple linear regression; SLR, simple linear regression; **bold figures**; statistically significant variables

Discussion

Cancer inflicts extensive physical, emotional, and social hardships on individuals and their families. Family caregivers navigate through an adjustment and acceptance phase in dealing with the illness, managing multiple responsibilities under significant stress levels, leading to physical, emotional, social, and financial strain, ultimately impacting their quality of life [1]. In an effort to evaluate the level of HRQoL and its determinants among family caregivers of cancer patients, this study revealed that the mean overall HRQoL score among participants was 52.7, with a standard deviation of 9.57. Among the four domains, the psychological domain exhibited the lowest mean score (47.82±16.61). Furthermore, the study identified predictor variables influencing HRQoL among the participants. Thus Being Spouse (β= -3.39; 95% CI: -6.49, -0.29), presence of chronic illness (β = -3.43; 95% CI: -5.56, -1.31), depression, (β = -2.55; 95% CI: -4.34, -0.75), anxiety (β = -3.27; 95% CI: -5.22, -1.32), and social support, (β = -3.61; 95% CI: -6.20, -1.02) were negatively associated with HRQoL. Psychological domain was the lowest which is in line with various evidences [18,34-36]. In contrast, findings from Turkey [37,38]. This difference could potentially be attributed to socio-cultural factors, variations in sample sizes, and the utilization of validated tools to measure HRQoL.

Participants who were spouses of the patients exhibited lower overall HRQoL compared to those who were siblings. This observation aligns with previous research conducted in Ethiopia [29] and China [12,39]. It is expected that the daily lives of cancer patients and their caregivers will be significantly disrupted. Furthermore, family caregivers are typically involved in all aspects of cancer care and management, requiring them to invest time, financial resources, and physical effort in navigating the complexities of cancer treatment.

This caregiving burden can negatively affect their physical and mental well-being. Thus, caring for a spouse with cancer can be overwhelming, leading to increased psychological stress and a diminished quality of life [39]. Additionally, spouses often bear the brunt of caregiving responsibilities and are less likely to receive external support, which can further impact their quality of life [29].

Regarding the existence of chronic illness, individuals with chronic conditions experienced a decrease in overall HRQoL compared to those without such conditions [21,37,40,41]. This phenomenon can be explained by the fact that physical well-being is a crucial aspect of quality of life for caregivers of oncology patients, given the physically and emotionally demanding nature of caregiving. As a result, healthcare professionals should take action by integrating caregivers into comprehensive cancer care protocols, encouraging them to prioritize their own health issues, promoting self-care practices, and offering guidance on accessing community and healthcare services [42].

Regarding the experience of depression and anxiety, individuals who exhibited these conditions had diminished overall HRQoL compared to those who did not. This finding aligns with several previous studies [22,40,43-46]. The reason behind this correlation may lie in the significant physical, emotional, and practical ramifications that a cancer diagnosis has on the lives of both patients and their families. The responsibilities placed on family caregivers of cancer patients are known to heighten caregiver strain, negatively impact mental and physical health, and worsen their quality of life [47]. Therefore, caregivers experiencing depression and anxiety should undergo proper screening and management to enhance patient care.

In terms of social support, caregivers who reported inadequate social support exhibited lower overall HRQoL compared to those who had strong social support. This observation is consistent with various studies [20,48,49]. The absence of adequate social support can signal both the level of direct care provided by caregivers to patients and their opportunities for relief from caregiving responsibilities. Therefore, enhancing both psychosocial support and available resources, such as home care services, could potentially enhance the quality of life for these caregivers.

Strength and limitation of study

This study is the first in Northwest Ethiopia, which indicated the level of health-related quality of life and its predictors. In addition using relative larger sample size on a multi-

facility setting and addressing the most likely potential determinant variables which can be an element for generalization. Despite this, the results presented in this study were self-reported, relying on the truthfulness of participants and may be exposed to recall bias. Additionally, substance use assessment is prone to social desirability bias. The cross-sectional nature of the study did not allow for a follow-up and made it difficult to establish a causal relationship. Even if the WHO BREF assessment tool is cross-culturally valid, it is not validated in the Ethiopian context.

Conclusion and recommendation

This study's findings suggest that caregivers of cancer patients may require improved psychological support, as evidenced by the lowest mean score observed in the psychological domain. Furthermore, there is a crucial need to prioritize efforts to bolster social support, particularly among caregivers in spousal relationships. Adequate clinical screening and follow-up are essential for individuals experiencing depression and anxiety to effectively manage these manifestations. Future research endeavors could delve deeper into exploring the causal relationship between health-related quality of life and various potential determinant variables.

Declaration

Consent for publication

NA

Availability of data and materials

The datasets used and analyzed during the current study are available.

Funding

This research work was not funded by any agent.

Competing interests

None declared.

Authors' contributions

FBT, EAM, AFD, FAT and SAW were participated in inception of idea, proposal development, data collection, analysis, and final write up. All authors approved the final manuscript.

Acknowledgments

The authors acknowledge Bahir Dar University and the study participants.

Reference

1. Guerra-Martín MD, Casado-Espinosa MDR, Gavira-López Y, Holgado-Castro C, López-Latorre I, et al. (2023) Quality of life in caregivers of cancer patients: a literature review. International Journal of Environmental Research and Public Health 20: 1570.
2. Moten A, Schafer D, Ferrari M (2014) Redefining global health priorities: Improving cancer care in developing settings. Journal of global health 4.
3. González Guerra A, Fonseca Fernández M, Valladares González A, López Angulo L (2017) Factores moduladores de resiliencia y sobrecarga en cuidadores principales de pacientes oncológicos avanzados. Revista Finlay 7: 26-32.
4. Zambrano-Domínguez EM, Guerra Martín MD (2012) Training of informal caregivers: Relationship to the time spent caring for dependent seniors over age 65. Aquichan, 12 (3), 241-251.
5. Organization WH (2016) WHOQOL User Manual. Geneva: World Health Organization, 1998.
6. Son KY, Lee CH, Park SM, Lee CH, Oh SI, et al. (2012) The factors associated with the quality of life of the spouse caregivers of patients with cancer: a cross-sectional study. Journal of palliative medicine 15: 216-224.
7. Amendola F, Oliveira MAdC, Alvarenga MRM (2011) Influence of social support on the quality of life of family caregivers while caring for people with dependence. Revista da Escola de Enfermagem da USP 45: 884-889.
8. Almutairi KM, Alodhayani AA, Alonazi WB, Vinluan JM (2017) Assessment of health-related quality of life among caregivers of patients with cancer diagnosis: a cross-sectional study in Saudi Arabia. Journal of religion and health 56: 226-237.
9. Li Q, Xu Y, Zhou H, Loke AY (2016) Factors influencing the health-related quality of life of Chinese advanced cancer patients and their spousal caregivers: a cross-sectional study. BMC Palliative Care 15: 1-14.
10. Meecharoen W, Sirapo-ngam Y, Monkong S, Oratai P, Northouse LL (2013) Factors influencing quality of life among family caregivers of patients with advanced cancer: A causal model. Pacific Rim International Journal of Nursing Research 17: 304-316.
11. Choi YS, Hwang SW, Hwang IC, Lee YJ, Kim YS, et al. (2016) Factors associated with quality of life among family caregivers of terminally ill cancer patients. Psycho-Oncology 25: 217-224.
12. Zhu P, Fu J-F, Wang B, Lin J, Wang Y, et al. (2014) Quality of life of male spouse caregivers for breast cancer patients in China. Asian Pacific Journal of Cancer Prevention 15: 4181-4185.
13. Lu L, Pan B, Sun W, Cheng L, Chi T, et al. (2010) Quality of life and related factors among cancer caregivers in China. Psychiatry and Clinical Neurosciences 64: 505-513.
14. Kim Y, Baker F, Spillers RL (2007) Cancer caregivers' quality of life: effects of gender, relationship, and appraisal. Journal of pain and symptom management 34: 294-304.
15. Thirumoorthy A, Devi PS, Thennarusu K (2016) Quality of life among the caregivers of persons living with cancer. IOSR J Humanities Soc Sci 21: 23-30.
16. Abdullah NN, Idris IB, Shamsuddin K, Abdullah NMA (2019) Health-related quality of life (HRQOL) of gastrointestinal cancer caregivers: the impact of caregiving. Asian Pacific Journal of Cancer Prevention: APJCP 20: 1191.
17. Leow MQ, Chan M-F, Chan SW (2014) Predictors of change in quality of life of family caregivers of patients near the end of life with advanced cancer. Cancer Nursing 37: 391-400.
18. Rha SY, Park Y, Song SK, Lee CE, Lee J (2015) Caregiving burden and the quality of life of family caregivers of cancer patients: the relationship and correlates. European Journal of Oncology Nursing 19: 376-382.

19. Clarijs ME, Oemrawsingh A, Bröker ME, Verhoef C, Lingsma H, et al. (2022) Quality of life of caregivers of breast cancer patients: a cross-sectional evaluation. Health and quality of life outcomes 20: 1-10.
20. Hsu T, Nathwani N, Loscalzo M, Chung V, Chao J, et al. (2019) Understanding caregiver quality of life in caregivers of hospitalized older adults with cancer. Journal of the American Geriatrics Society 67: 978-986.
21. Navied U, Arshad S, Ahmed U, Hameed A (2020) The Quality of Life amongst Caregivers of Cancer Patients at Tertiary Care Centers in Lahore, Pakistan. Biomedica 36.
22. Odeny HA, Arudo JO, Psusma TS (2023) Assessment of Socioeconomic and Psychological Factors Predicting Health-Related Quality of Life among Primary Caregivers of Cancer Patients in Kakamega County. Evidence-Based Nursing Research 6: 1-13.
23. Rostami M, Abbasi M, Soleimani M, Moghaddam ZK, Zeraatchi A (2023) Quality of life among family caregivers of cancer patients: an investigation of SF-36 domains. BMC psychology 11: 445.
24. Kroenke K, Spitzer RL, Williams JB, Monahan PO, Löwe B (2007) Anxiety disorders in primary care: prevalence, impairment, comorbidity, and detection. Annals of internal medicine 146: 317-325.
25. Gelaye B, Williams MA, Lemma S, Deyessa N, Bahretibeb Y, et al. (2013) Validity of the patient health questionnaire-9 for depression screening and diagnosis in East Africa. Psychiatry research 210: 653-661.
26. Group W (1998) Development of the World Health Organization WHOQOL-BREF quality of life assessment. Psychological medicine 28: 551-558.
27. Abiola T, Udofia O, Zakari M (2013) Psychometric properties of the 3-item oslo social support scale among clinical students of Bayero University Kano, Nigeria. Malaysian Journal of Psychiatry 22: 32-41.
28. Group WAW (2002) The alcohol, smoking and substance involvement screening test (ASSIST): development, reliability and feasibility. Addiction 97: 1183-1194.
29. Yihedego E, Aga F, Gela D, Boka A (2020) Quality of life and associated factors among family caregivers of adult cancer patients in Addis Ababa, Ethiopia. Cancer Management and Research: 10047-10054.
30. Skevington SM, Lotfy M, O'Connell KA (2004) The World Health Organization's WHOQOL-BREF quality of life assessment: psychometric properties and results of the international field trial. A report from the WHOQOL group. Quality of life Research 13: 299-310.
31. Mas-Expósito L, Amador-Campos JA, Gómez-Benito J, Lalucat-Jo L (2011) The World Health Organization quality of life scale brief version: a validation study in patients with schizophrenia. Quality of life Research 20: 1079-1089.
32. Koboto DD, Deribe B, Gebretsadik A, Ababi G, Bogale N, et al. (2020) Quality of life among breast cancer patients attending Hawassa University comprehensive specialized hospital cancer treatment center. Breast cancer: targets and therapy: 87-95.
33. Andrade EM, Geha LM, Duran P, Suwwan R, Machado F, et al. (2016) Quality of life in caregivers of ADHD children and diabetes patients. Frontiers in Psychiatry 7: 127.
34. Ovayolu Ö, Ovayolu N, Tuna D, Serçe S, Sevinç A, et al. (2014) Quality of life of caregivers: A cross-sectional study. International journal of nursing practice 20: 424-432.
35. Valluri V (2023) Assessment of Quality of Life of Caregivers of Persons living with Cancers: a tertiary health centre-based cross-sectional study. Qeios.
36. Carter G, Lewin T, Rashid G, Adams C, Clover K (2008) Computerised assessment of quality of life in oncology patients and carers. Psycho-Oncology 17: 26-33.
37. Hacialioglu N, Özer N, Erdem N, Erci B (2010) The quality of life of family caregivers of cancer patients in the East of Turkey. European Journal of Oncology Nursing 14: 211-217.
38. Alptekin S, Gönüllü G, Yücel İ, Yarış F (2010) Characteristics and quality of life analysis of caregivers of cancer patients. Medical Oncology 27: 607-617.

39. Wang C, Wacharasin C, Hengudomsub P (2023) Self-efficacy as Mediators of the Association Between Dyadic Coping and Quality of Life among Spousal Caregiver of Patients with Cervical Cancer. Asia-Pacific Journal of Oncology Nursing: 100354.
40. Doubova SV, Infante-Castañeda C (2016) Factors associated with quality of life of caregivers of Mexican cancer patients. Quality of Life Research 25: 2931-2940.
41. Chow WL, Tan S-M, Aung KCY, Chua SYN, Sim HC (2020) Factors influencing quality of life of Asian breast cancer patients and their caregivers at diagnosis: perceived medical and psychosocial needs. Singapore Medical Journal 61: 532.
42. Glajchen M. Physical well-being of oncology caregivers: an important quality-of-life domain; 2012. Elsevier. pp. 226-235.
43. García-Carmona M, García-Torres F, Jacek Jabłoński M, Gomez Solis A, Jaén-Moreno MJ, et al. (2021) The influence of family social support on quality of life of informal caregivers of cancer patients. Nursing Open 8: 3411-3419.
44. Ayabakan-Cot D, Ates E, Kurt B, Nazlican E, Akbaba M (2017) Investigation of depression and quality of life factors in cancer patients' caregivers. Age (years) 18: 91.
45. Douglas SL, Daly BJ (2013) The impact of patient quality of life and spirituality upon caregiver depression for those with advanced cancer. Palliative & supportive care 11: 389-396.
46. Garcia-Torres F, Jabłoński MJ, Gómez-Solís A, Moriana JA, Jaén-Moreno M, et al. (2022) Anxiety, depression and quality of life: a longitudinal study involving cancer patient-caregiver dyads. Health Psychology Report 9.
47. Song JI, Shin DW, Choi JY, Kang J, Baik YJ, et al. (2011) Quality of life and mental health in family caregivers of patients with terminal cancer. Supportive Care in Cancer 19: 1519-1526.
48. Lee YJ, Kim JE, Choi YS, Hwang IC, Hwang SW, et al. (2016) Quality of life discordance between terminal cancer patients and family caregivers: a multicenter study. Supportive Care in Cancer 24: 2853-2860.
49. Eom CS, Shin DW, Kim SY, Yang HK, Jo HS, et al. (2013) Impact of perceived social support on the mental health and health-related quality of life in cancer patients: results from a nationwide, multicenter survey in South Korea. Psycho-Oncology 22: 1283-1290.

Click here to access/download
Supporting Information
PLOSOne_Human_Subjects_Research_Checklist.docx

Click here to access/download
Supporting Information
STROBE Checklist.docx
