# Supplementary material for: Health-related quality of life and associated factors among family caregivers of patients with cancer in oncologic centers of Northwest Ethiopia
Source: PLoS One. 2024 May 31;19(5):e0304392. doi: 10.1371/journal.pone.0304392 (PMC11142700; doi:10.1371/journal.pone.0304392)
Supplement: Attachment — Submitted filename: Response to Reviewers.docx [file pone.0304392.s006.docx]

**Responses to the review’s comments**

**Dear PLOS ONE editorial team,**

Thank you for giving us the opportunity to submit a revised draft of the manuscript, and we would also like to thank you for your crucial comments on our paper **(**Manuscript ID: **PONE-D-24-10340**). We are very concerned and have combined all the suggested comments provided, which we believe strengthen our paper, and we hope this will render our paper eligible for consideration for publication in your reputed journal. We appreciate the time and effort that you and the reviewers dedicated to providing feedback on our manuscript and are grateful for the insightful comments and valuable improvements to our paper.

The authors would like to inform you that we have addressed the comments and recommendations made by both reviewers and the editor, point by point. In addition, throughout our revision, we made our best corrections too. All changes in the revised manuscript are highlighted using tracking changes within the manuscript. Please see below, in blue, for a point-by-point response to the reviewers’ comments and concerns. All page numbers refer to the revised manuscript file with tracked changes.

**Comments from the editor:**

Journal Requirements:

When submitting your revision, we need you to address these additional requirements.

**#1…...**Please ensure that your manuscript meets PLOS ONE's style requirements, including those for file naming.

**Author response:** Thank you for your recommendationstoadhere PLOS ONE’s style of requirements. We have ensured that the manuscript, its name, figures, and tables are per the journal requirements.

**#2…..** We noticed you have some minor occurrence of overlapping text with the following previous publication(s), which needs to be addressed:

- <https://doi.org/10.1371/journal.pone.0281742>
- doi:10.1136/bmjopen-2023-074112

In your revision ensure you cite all your sources (including your own works), and quote or rephrase any duplicated text outside the methods section. Further consideration is dependent on these concerns being addressed.

**Author response:**

**#3…...** Please provide a complete Data Availability Statement in the submission form, ensuring you include all necessary access information or a reason for why you are unable to make your data freely accessible. If your research concerns only data provided within your submission, please write "All data are in the manuscript and/or supporting information files" as your Data Availability Statement.

**Author response:** Thank you very much for your request to revise the data availability statement. Based on your recommendation, we have revised it and stated in the main document as well as in the cover letter as “All necessary files are available in the manuscript including the datasets as supporting files (S1 File. Dataset and S2 File. Dataset)”.

**#4…..**Please include captions for your Supporting Information files at the end of your manuscript, and update any in-text citations to match accordingly. Please see our Supporting Information guidelines for more information: <http://journals.plos.org/plosone/s/supporting-information>.

**Author response:** Thank you very much for the comment and we have added the caption for all supporting information.

**#5……**Please review your reference list to ensure that it is complete and correct. If you have cited papers that have been retracted, please include the rationale for doing so in the manuscript text, or remove these references and replace them with relevant current references. Any changes to the reference list should be mentioned in the rebuttal letter that accompanies your revised manuscript. If you need to cite a retracted article, indicate the article’s retracted status in the References list and also include a citation and full reference for the retraction notice.

**Author response:** Thank you very much for your suggestion and we would like to assure you that retracted reference was not used in the manuscript.

**Response to Reviewers’ comments**

**Reviewer 1**

**#1…...**Title: please remove "2023"

**Author response:** Thank you for your comment and we have taken it.

**#2…...** Results: For Table 1 and 6, please put currency reference (e.g I ET birr= x USD)

**Author response:** Thank you very much for your recommendation and we have included the equivalent currency in USD.

**#3…..**Table 2: please correct the spelling for Hodgkin's lymphoma

**Author response:** Thank you very much for identifying the typing error and it is corrected.

**#4…...**Table 2: please do not use * for indicating the mean, please write Mean (SD) = the actual value

**Author response** Thank you very much for your suggestion and correction was made.

**#5…...**Under the following sub topic (Factors associated with health related quality of life among family caregivers of cancer patients), please rephrase the first sentence "After adjusting for multiple linear regression analysis" to After adjusting for confounders by applying multiple linear regression analysis,

**Author response:**  Thank you very much for recommending the way of writing and amendment was made.

**#6…...**Conclusion and recommendation: Please summarise your major findings first: the level of HRQoL, folowed with the associated factors before suggesting with screening etc.

**Author response** Thank you very much for your insight and we have revised the conclusion and recommendation section to make it clear.

**Reviewer 2:**

**#1…...** Comma here

**Author response:** Thank you very much for your comment and we have added the comma.

**#2…...** What is this number for?

**Author response:** Thank you very much for identifying the error made and it is corrected.
